# Supplementary material for: DNA methylation changes during long-term in vitro cell culture are caused by epigenetic drift
Source: Commun Biol. 2021 May 19;4:598. doi: 10.1038/s42003-021-02116-y (PMC8134454; doi:10.1038/s42003-021-02116-y)
Supplement: Supplementary file 2 — Supplementary Information [file 42003_2021_2116_MOESM2_ESM.pdf]

Supplemental Material

DNA methylation changes during long-term *in vitro* cell culture are caused by epigenetic drift

Julia Franzen, Theodoros Georgomanolis, Anton Selich, Chao-Chung Kuo, Reinhard Stöger, Lilija Brant, Melita Sara Mulabdić, Eduardo Fernandez-Rebollo, Clara Grezella, Alina Ostrowska, Matthias Begemann, Miloš Nikolić, Björn Rath, Anthony D. Ho, Michael Rothe, Axel Schambach, Argyris Papantonis, Wolfgang Wagner

Contents

Supplemental Figure S1: Growth curves and long-term culture-associated changes in cell preparations. 2
Supplemental Figure S2: DNAm changes of different CpG subsets are reset at day 20 during reprogramming into iPSCs. 3
Supplemental Figure S3: Variations of DNAm patterns and estimation of Shannon index. 4
Supplemental Figure S4: Analysis of hairpin BBA-Seq. 5
Supplemental Figure S5: Circular chromatin conformation capture in cis contacts. 6
Supplemental Figure S6: Analysis of CTCF binding in early and late passage cells. 7
Supplemental Table S1. Illumina 450k BeadChip datasets. 8
Supplemental Table S2. Thirty selected long-term culture-associated CpGs 10
Supplemental Table S3. Pyrosequencing primers 11
Supplemental Table S4. Pyrosequencing results of training dataset 12
Supplemental Table S5. BBAseq primers 13
Supplemental Table S6. Hairpin linkers 14
Supplemental Table S7. Hairpin primers 14
Supplemental Table S8. 4C primers 15
Supplemental References 15

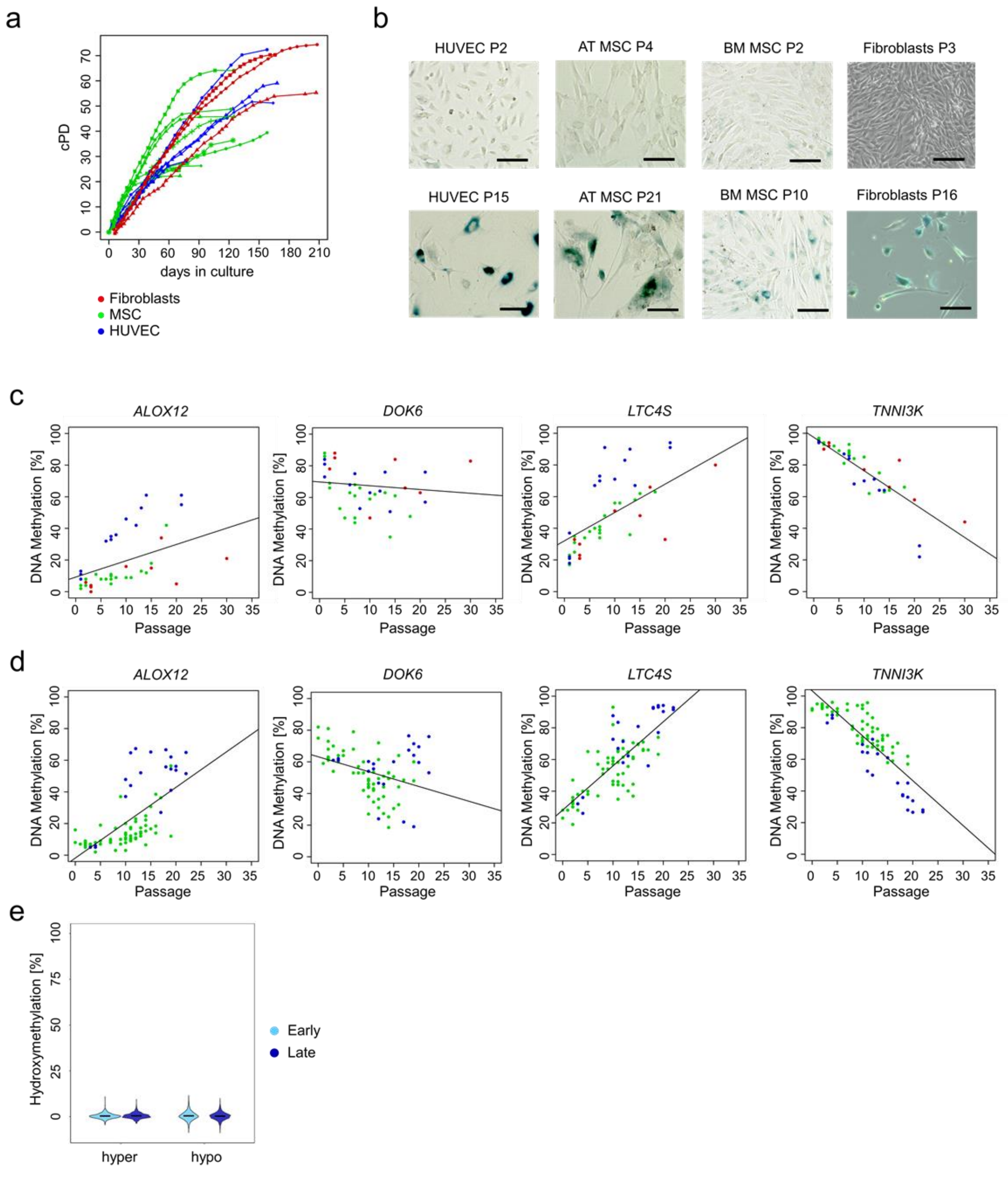

Supplemental Figure S1: Growth curves and long-term culture-associated changes in cell preparations.

a) Growths curves of the cell preparations of the training set (each dot represents a passage; cPD = cumulative population doublings). **b)** Representative images of senescence-associated beta galactosidase staining (AT = adipose-tissue-derived, BM = bone-marrow-derived, P = passage, size bar = 100 µm). DNAm levels were measured by pyrosequencing in the training **(c)**, and validation set **(d)**. DNAm at all of the four long-term culture-associated CpGs correlated with passage numbers of cell preparations. **e)** Hydroxymethylation was analyzed with the TrueMethyl Array kit for three MSC donors in early (passage 4) and late (passage 10) passages. The plots depict hydroxymethylation (5hmC; %) at 646 hyper- and 2,442 hypomethylated culture-associated CpGs. Neither hyper- nor hypomethylated sites show high values of hydroxymethylation.

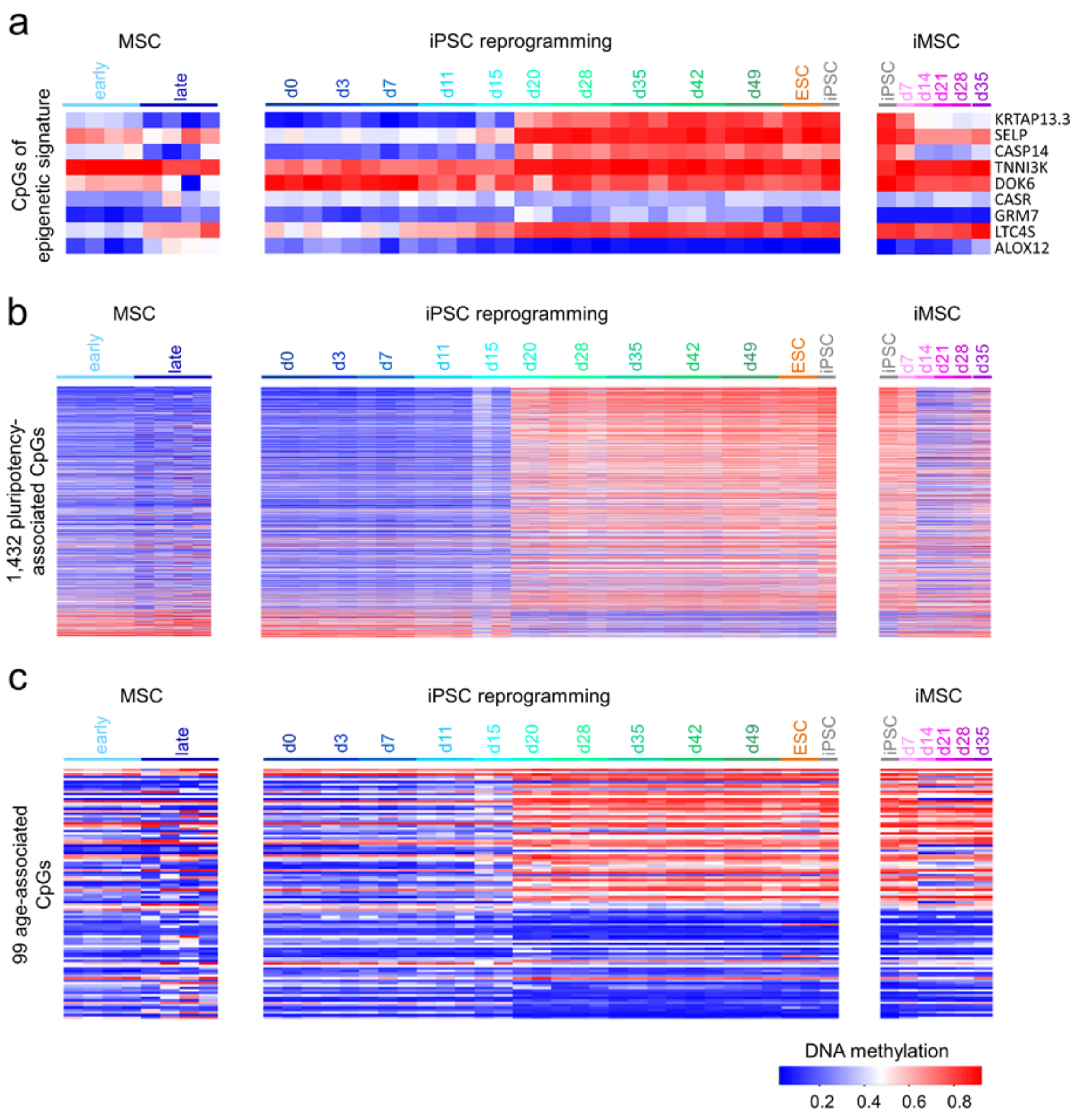

Supplemental Figure S2: DNAm changes of different CpG subsets are reset at day 20 during reprogramming into iPSCs.

DNAm levels at **a)** 9 CpGs of our epigenetic signatures [1], **b)** 1,432 pluripotency associated CpG sites [2], and **c)** 99 age-associated CpGs of our previously described age-predictor for blood samples [3] are depicted in MSCs of early (passage 2) and late passages (passage 7 to 16; GSE37067) [4], during reprogramming of fibroblasts into iPSCs (GSE54848) [5], and re-differentiation of iPSCs towards MSCs (iMSCs; GSE54767) [6].

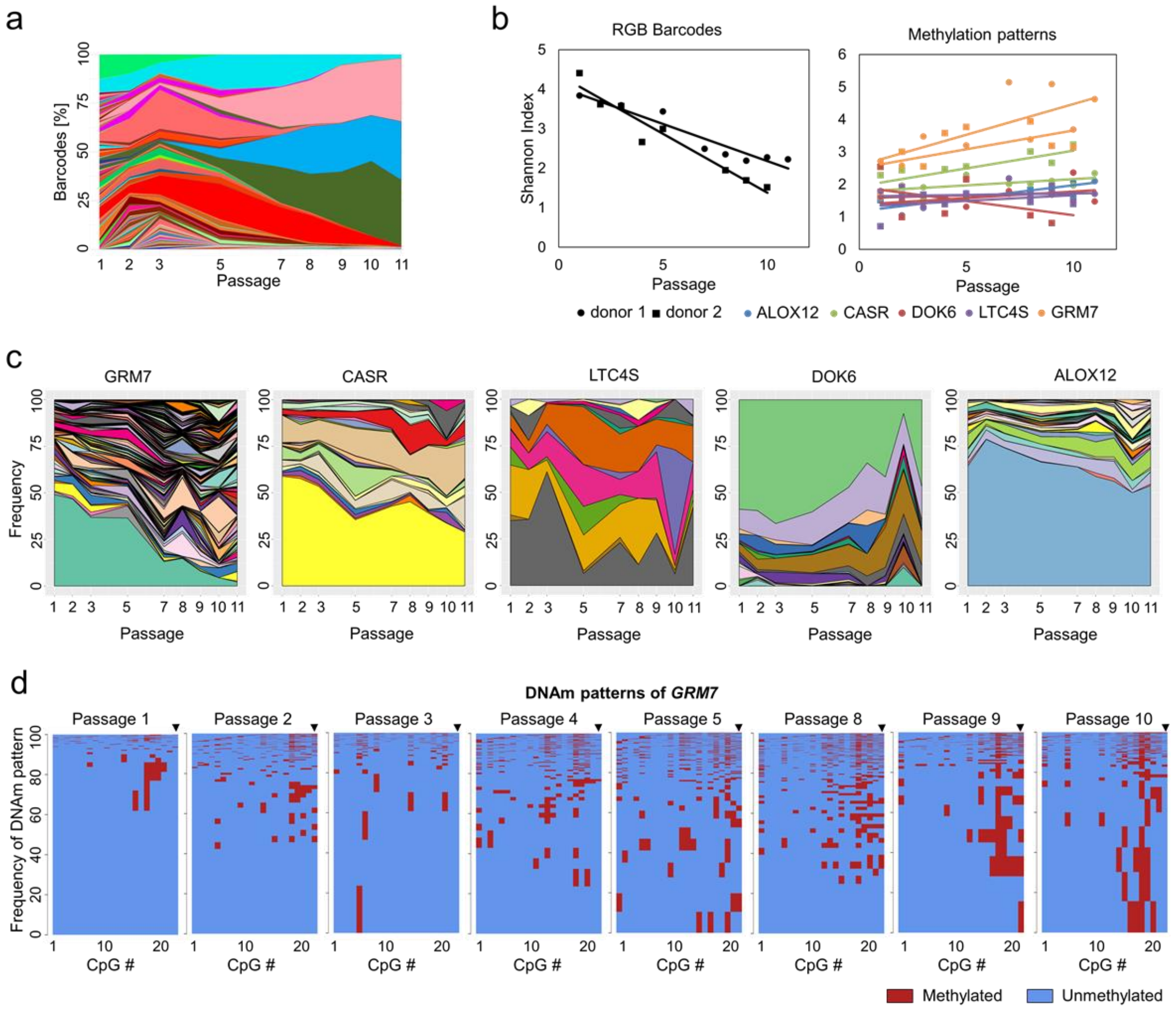

Supplemental Figure S3: Variations of DNAm patterns and estimation of Shannon index.

a) Deep sequencing analysis of random barcodes demonstrates that MSCs of the second preparation become oligoclonal at later passages (in analogy to Figure 3a). **b)** Shannon index of RGB barcodes in the two UC-MSC donors and of the DNAm patterns in five different amplicons of the two donors. **c)** Frequencies of different DNAm patterns within neighboring CpGs for the second MSC preparation (in analogy to Figure 3c). **d)** Individual DNAm patterns are exemplarily depicted for 22 neighboring CpGs of *GRM7* of UC-MSC donor 2 (corresponding to Figure 3). The frequency of DNAm patterns resembles the percentage of corresponding reads in deep sequencing analysis. The height of each pattern corresponds to the frequency of this specific DNAm pattern, while the sum of all pattern frequencies adds up to 100%.

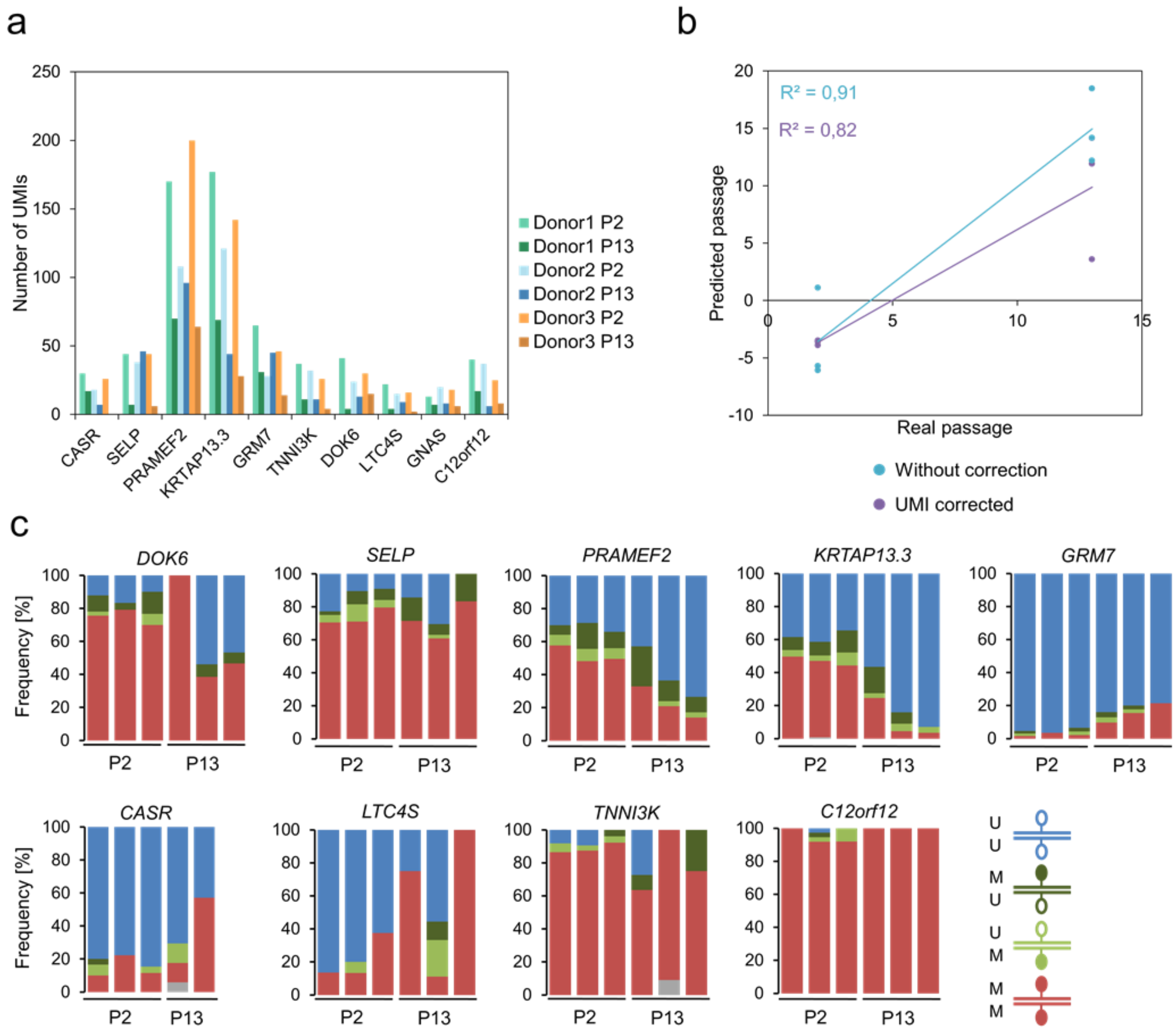

Supplemental Figure S4: Analysis of hairpin BBA-Seq.

a) Number of different unique molecular identifiers (UMIs) within hairpin loops detected for each amplicon. **b)** Passage predictions based on eight culture-associated CpGs either without or with consideration of different UMIs. **c)** Frequency of homo- and hemimethylation at culture-expansion-associated CpG sites that were used for predictions of passage numbers and at a highly methylated region (*C12orf12*). Grey colored regions resemble sequencing errors at one of the complementary CpG sites. One late passage donor of *CASR* is missing due to failed sequencing.

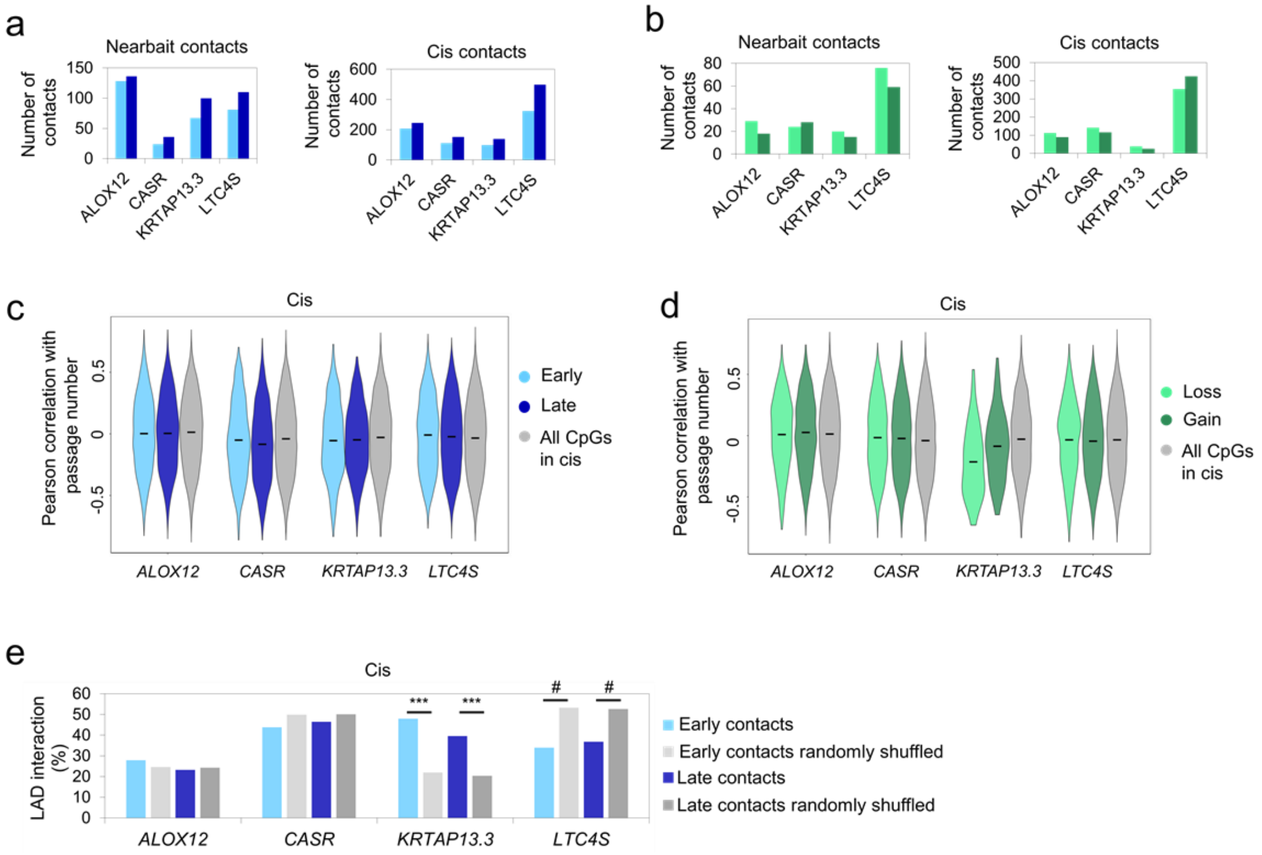

Supplemental Figure S5: Circular chromatin conformation capture in cis contacts.

a) Numbers of highly interacting regions called by 4Cker within nearbait (10 MB around the bait locus of interest) and cis contacts (same chromosome) of the four culture expansion associated CpGs. The histograms depict shared contacts of two donors for early passage and two donors for late passage, and they were similar at early and late passages. **b)** Numbers of significant differential contacts (p-value < 0.05 by the FDR corrected 4Cker analysis) between early and late passages of all four regions in nearbait and cis contacts. **c)** Violin plots of the Pearson correlation of DNAm with passage numbers of all CpGs within the cis contacts of early passaged cells (light blue) or late passaged cells (dark blue) in comparison to all CpGs of the cis region (grey). Means are depicted by black bars and show that the subsets of CpGs within the highly interacting sites do not deviate from the overall mean of the cis region. **d)** In analogy to c) the violin plots depict the Pearson correlation for CpGs within the significantly differential contacts of the cis region in comparison to all CpGs of the cis region. **e)** Enrichment of lamina-associated domains (LADs) of a publicly available dataset of human fibroblasts [7] within high interacting sites of the four baits in cis compared to the mean interaction frequency of random background regions of the same size, which have been shuffled along the cis chromosome 1000 times. Significance was tested by Fisher's exact test (* $p < 0.05$; ** $p < 0.01$; *** $p < 0.001$; # $p < 0.0001$).

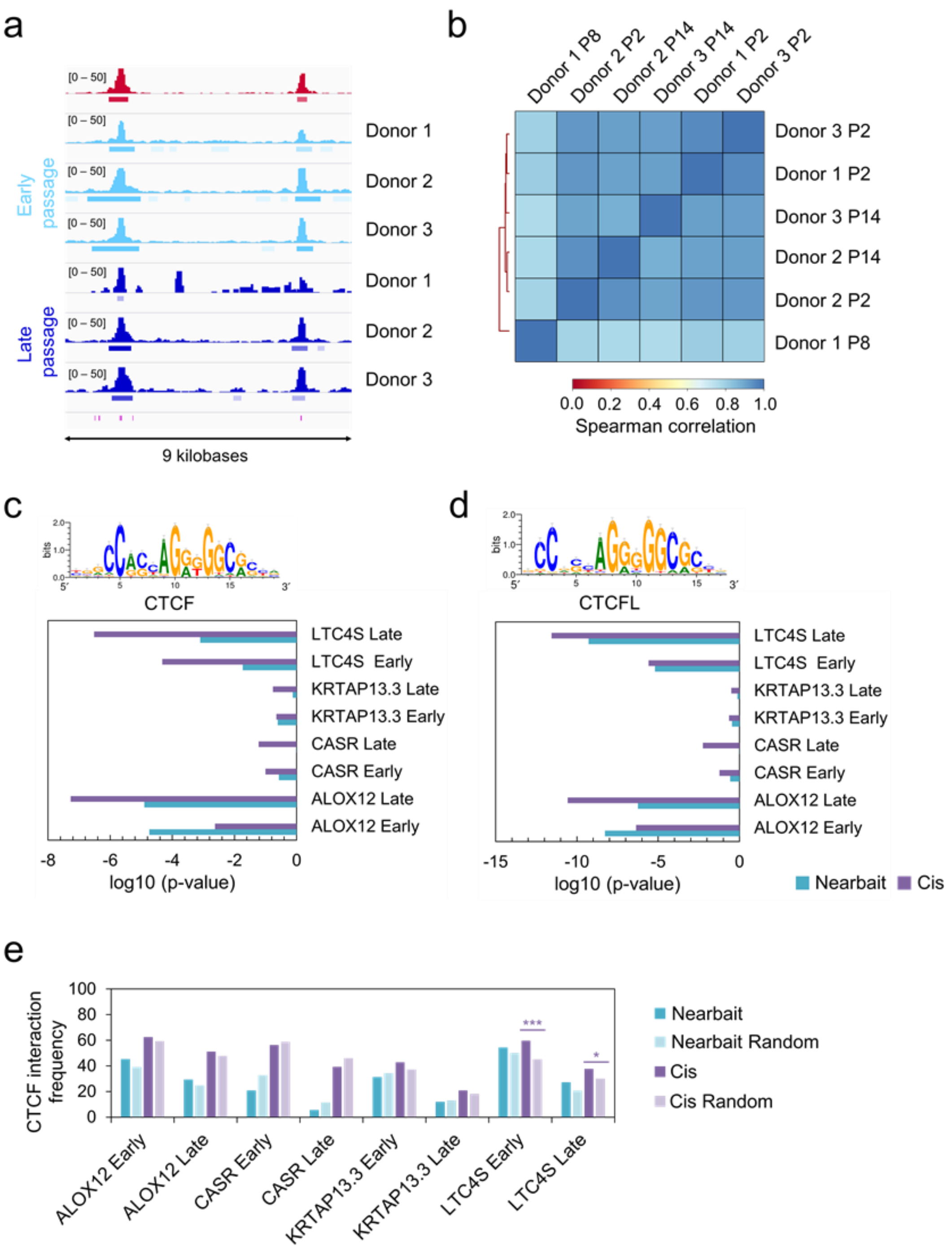

Supplemental Figure S6: Analysis of CTCF binding in early and late passage cells. a) Representative Integrative Genomics Viewer (IGV) overview of ChIP-seq peaks (chr5: 179221657 – 179230585; hg19, close to *LTC4S*) in early (P2, light blue) and late (P8 – P14, dark blue) passages. Peaks are associated to predicted CTCF motifs (violet lines at the bottom) and are reproducible in several donors, both in early and late passages as well as in a publicly available dataset (red, embryonic stem cell derived MSCs) [8]. **b)** Spearman correlation of normalized CTCF ChIP-seq peaks reveals high correlation for all samples irrespective of their passage number. **c, d)** Enrichment analysis of predicted motif binding sites for CTCF (c) and CTCFL (d) in high interacting regions of the 4C experiment. P-values were calculated with the RGT motif enrichment tool. Significant enrichment was particularly observed for the hypermethylated CpGs in *LTC4S* and *ALOX12*. **e)** Enrichment of CTCF ChIP-seq peaks within high interacting sites of the four baits in the 4C experiment compared to random background regions. To this end, CTCF binding peaks were further divided into early or late CTCF peaks, derived from corresponding early or late passaged samples respectively. Significance was tested by Fisher's exact test (* $p < 0.05$; ** $p < 0.01$; *** $p < 0.001$).

Supplemental Table S1. Illumina 450k BeadChip datasets.

Geo accession	GSE number	cell type	passage	Publication
**GSM1004625**	GSE40927	fibroblast	4	Kurian, et al. [9]
**GSM1004626**	GSE40927	fibroblast	5	Kurian, et al. [9]
**GSM1004627**	GSE40927	fibroblast	14	Kurian, et al. [9]
**GSM1004643**	GSE40927	HUVEC	4	Kurian, et al. [9]
**GSM1004644**	GSE40927	HUVEC	4	Kurian, et al. [9]
**GSM1004645**	GSE40927	HUVEC	17	Kurian, et al. [9]
**GSM1027664**	GSE41933	MSC	2	Reinisch, et al. [10]
**GSM1027665**	GSE41933	MSC	2	Reinisch, et al. [10]
**GSM1027666**	GSE41933	MSC	2	Reinisch, et al. [10]
**GSM1027667**	GSE41933	MSC	2	Reinisch, et al. [10]
**GSM1027668**	GSE41933	MSC	2	Reinisch, et al. [10]
**GSM1027669**	GSE41933	MSC	2	Reinisch, et al. [10]
**GSM1027670**	GSE41933	MSC	2	Reinisch, et al. [10]
**GSM1027671**	GSE41933	MSC	2	Reinisch, et al. [10]
**GSM1027672**	GSE41933	MSC	2	Reinisch, et al. [10]
**GSM1027673**	GSE41933	fibroblast	2	Reinisch, et al. [10]
**GSM1027674**	GSE41933	fibroblast	2	Reinisch, et al. [10]
**GSM1027675**	GSE41933	fibroblast	2	Reinisch, et al. [10]
**GSM853409**	GSE37066	MSC	2	Koch, et al. [4]
**GSM853410**	GSE37066	MSC	2	Koch, et al. [4]
**GSM853411**	GSE37066	MSC	2	Koch, et al. [4]
**GSM853412**	GSE37066	MSC	2	Koch, et al. [4]
**GSM853413**	GSE37066	MSC	3	Koch, et al. [4]
**GSM909610**	GSE37066	MSC	7	Koch, et al. [4]
**GSM909612**	GSE37066	MSC	12	Koch, et al. [4]
**GSM909614**	GSE37066	MSC	16	Koch, et al. [4]
**GSM909616**	GSE37066	MSC	14	Koch, et al. [4]
**GSM909618**	GSE37066	MSC	14	Koch, et al. [4]
**GSM3230133**	GSE116375	MSC	4	this study
**GSM3230134**	GSE116375	MSC	4	this study
**GSM3230135**	GSE116375	MSC	4	this study
**GSM3230136**	GSE116375	MSC	10	this study
**GSM3230137**	GSE116375	MSC	10	this study
**GSM3230138**	GSE116375	MSC	10	this study
**GSM2340959**	GSE87797	MSC	2	Fernandez-Rebollo, et al. [11]
**GSM2340960**	GSE87797	MSC	2	Fernandez-Rebollo, et al. [11]
**GSM2340961**	GSE87797	MSC	2	Fernandez-Rebollo, et al. [11]
**GSM2340962**	GSE87797	MSC	2	Fernandez-Rebollo, et al. [11]
**GSM2340963**	GSE87797	MSC	2	Fernandez-Rebollo, et al. [11]
**GSM2340964**	GSE87797	MSC	2	Fernandez-Rebollo, et al. [11]
**GSM2340965**	GSE87797	MSC	2	Fernandez-Rebollo, et al. [11]
**GSM2340966**	GSE87797	MSC	2	Fernandez-Rebollo, et al. [11]
**GSM2340967**	GSE87797	MSC	2	Fernandez-Rebollo, et al. [11]
**GSM2340968**	GSE87797	MSC	2	Fernandez-Rebollo, et al. [11]
**GSM2340969**	GSE87797	MSC	2	Fernandez-Rebollo, et al. [11]

**GSM2340970**	GSE87797	MSC	2	Fernandez-Rebollo, et al. [11]
**GSM1347975**	GSE55888	MSC	4	Schellenberg, et al. [12]
**GSM1347978**	GSE55888	MSC	4	Schellenberg, et al. [12]
**GSM1347981**	GSE55888	MSC	4	Schellenberg, et al. [12]
**GSM1347984**	GSE55888	MSC	4	Schellenberg, et al. [12]
**GSM1347987**	GSE55888	MSC	4	Schellenberg, et al. [12]
**GSM1347989**	GSE55888	MSC	4	Schellenberg, et al. [12]
**GSM1347991**	GSE55888	MSC	4	Schellenberg, et al. [12]
**GSM1347993**	GSE55888	MSC	4	Schellenberg, et al. [12]
**GSM2186934**	GSE82234	HUVEC	4	Franzen, et al. [13]
**GSM2186935**	GSE82234	HUVEC	4	Franzen, et al. [13]
**GSM2186936**	GSE82234	HUVEC	4	Franzen, et al. [13]
**GSM3230129**	GSE116375	HUVEC	10	this study
**GSM3230130**	GSE116375	HUVEC	15	this study
**GSM3230131**	GSE116375	HUVEC	12	this study
**GSM3230132**	GSE116375	HUVEC	8	this study
**GSM2186937**	GSE82234	HUVEC	20	Franzen, et al. [13]
**GSM2186938**	GSE82234	HUVEC	18	Franzen, et al. [13]
**GSM2186939**	GSE82234	HUVEC	13	Franzen, et al. [13]

Supplemental Table S2. Thirty selected long-term culture-associated CpGs

Illumina ID	Pearson (R)	R^2	Slope (m)
**cg25281820**	-0.87786992	0.770655597	-0.02079236
**cg03421657**	-0.86871368	0.754663451	-0.02217539
**cg21567022**	-0.86815882	0.753699738	-0.03263046
**cg10588720**	-0.86669937	0.751167791	-0.02130874
**cg16470423**	-0.86548811	0.749069674	-0.02626191
**cg23054188**	-0.86328129	0.74525459	-0.02776338
**cg05264232**	-0.86248453	0.743879567	-0.02051906
**cg07403610**	-0.86147656	0.742141858	-0.024732
**cg17625256**	-0.8603098	0.740132952	-0.0238157
**cg00063346**	-0.85976139	0.73918965	-0.02255066
**cg25968937**	-0.85690853	0.734292227	-0.03551924
**cg00791548**	-0.85496971	0.730973203	-0.02079242
**cg07566463**	-0.85404073	0.729385561	-0.02079289
**cg16819051**	-0.85340441	0.728299093	-0.02279132
**cg08732456**	-0.85092846	0.724079248	-0.02796559
**cg04682775**	0.80592194	0.649510173	0.02106277
**cg19443920**	0.80854957	0.653752415	0.02610184
**cg17180284**	0.80937209	0.655083181	0.02006929
**cg24628744**	0.80950526	0.655298762	0.02028689
**cg06221449**	0.81021837	0.656453811	0.02305057
**cg01815912**	0.81744955	0.668223773	0.02761235
**cg19759135**	0.81834739	0.669692446	0.02261348
**cg05054998**	0.81942121	0.671451112	0.03632707
**cg23602058**	0.82959135	0.688221809	0.02256118
**cg26683398**	0.84259943	0.709973806	0.02118855
**cg03762994**	0.84439666	0.713005722	0.02743842
**cg02717339**	0.84612683	0.715930609	0.0210322
**cg27456203**	0.85926672	0.738339289	0.02156726
**cg11394785**	0.86553634	0.749153148	0.02060183
**cg04065086**	0.8684504	0.754206094	0.02188371

Supplemental Table S3. Pyrosequencing primers

gene name	primer	Sequence 5’ -> 3’
ALOX12	forward	GGGGTTATTTTTAATTTTTAAAGGAT
ALOX12	reverse	Biotin - AAAACACAACCAATCCCCACAA
ALOX12	sequencing	TTATTTTTAATTTTTAAAGGA
DOK6	forward	Biotin-ATGAGAAAATTGAGATATAATTTTATTTAGGAAATAG
DOK6	reverse	CACCTTTTCTTCTAAAATCTACAAATCCC
DOK6	sequencing	TCACACTCAAAATCTAACCTA
LTC4S	forward	TTTTAGGGTTTTGTAGATTTTTATATTATGTTGGAGTTAG
LTC4S	reverse	Biotin - CACCCAAAAAACCTTAAACAAATTTCC
LTC4S	sequencing	AATTTTTGTTAAATTTTTTTTT
TNNI3K	forward	Biotin- ATTTTGTGGTTTTTTATAATGTTTTAGGAGTGTGATAA
TNNI3K	reverse	CCATAATCACTTTATTCACTACATCACCAATACCCATTC
TNNI3K	sequencing	CAATAAAATACCTAACATAATACT

Supplemental Table S4. Pyrosequencing results of training dataset

Sample	*ALOX12*	*DOK6*	*LTC4S*	*TNNI3K*	Real passage	Predicted passage
**AT MSC 1**	0.04	0.88	0.23	0.95	1	3
**AT MSC 1**	0.09	0.66	0.48	0.84	8	8
**AT MSC 1**	0.18	0.61	0.62	0.64	15	16
**AT MSC 2**	0.04	0.86	0.23	0.97	1	2
**AT MSC 2**	0.09	0.59	0.56	0.85	10	8
**AT MSC 2**	0.42	0.48	0.63	0.66	18	12
**AT MSC 3**	0.02	0.88	0.17	0.96	1	2
**AT MSC 3**	0.05	0.61	0.39	0.89	7	6
**AT MSC 3**	0.12	0.35	0.58	0.63	14	16
**Fibroblast**	0.06	0.78	0.33	0.9	2	5
**Fibroblast**	0.16	0.47	0.51	0.77	10	10
**Fibroblast**	0.34	0.66	0.66	0.83	17	7
**Fibroblast**	0.04	0.85	0.3	0.92	3	4
**Fibroblast**	0.2	0.85	0.59	0.32	37	28
**Fibroblast**	0.03	0.88	0.23	0.94	3	3
**Fibroblast**	0.15	0.84	0.48	0.66	15	14
**Fibroblast**	0.21	0.83	0.8	0.44	30	24
**Fibroblast**	0	0.85	0.21	0.92	3	4
**Fibroblast**	0.05	0.63	0.33	0.58	20	18
**HUVEC 1**	0.13	0.73	0.37	0.94	1	3
**HUVEC 1**	0.36	0.53	0.91	0.68	8	14
**HUVEC 1**	0.61	0.51	0.67	0.64	14	11
**HUVEC 2**	0.13	0.81	0.22	0.94	1	2
**HUVEC 2**	0.32	0.68	0.67	0.87	6	5
**HUVEC 2**	0.46	0.63	0.71	0.7	10	11
**HUVEC 3**	0.08	0.81	0.21	0.95	1	2
**HUVEC 3**	0.33	0.75	0.7	0.84	7	7
**HUVEC 3**	0.42	0.64	0.83	0.71	12	11
**HUVEC 3**	0.55	0.57	0.94	0.29	21	27
**HUVEC 4**	0.11	0.84	0.18	0.95	1	2
**HUVEC 4**	0.35	0.68	0.73	0.86	7	6
**HUVEC 4**	0.53	0.76	0.9	0.64	13	13
**HUVEC 4**	0.61	0.76	0.91	0.22	21	29
**BM MSC 1**	0.08	0.66	0.31	0.93	2	4
**BM MSC 1**	0.11	0.47	0.41	0.93	7	4
**BM MSC 2**	0.04	0.66	0.25	0.94	2	3
**BM MSC 2**	0.08	0.44	0.34	0.87	7	6
**BM MSC 2**	0.13	0.63	0.36	0.75	13	10
**BM MSC 3**	0.08	0.69	0.35	0.93	2	4
**BM MSC 3**	0.08	0.63	0.4	0.81	6	9
**BM MSC 3**	0.09	0.62	0.56	0.62	11	17
**BM MSC 2**	0.1	0.68	0.37	0.88	7	6
**BM MSC 3**	0.08	0.47	0.38	0.89	5	6
**BM MSC 1**	0.11	0.53	0.34	0.92	4	4

Methylation values of single CpGs are depicted as β-values ranging from 0 to 1.

Supplemental Table S5. BBAseq primers

gene name	primer	Sequence 5’ -> 3’
ALOX12	forward	CTCTTTCCCTACACGACGCTCTTCCGATCTGGGGTTATTTTTAATTTTTAAAGGAT
ALOX12	reverse	CTGGAGTTCAGACGTGTGCTCTTCCGATCTAAAACACAACCAATCCCCACAA
DOK6	forward	CTCTTTCCCTACACGACGCTCTTCCGATCTATGAGAAAATTGAGATATAATTTTATTTAGGAAATAG
DOK6	reverse	CTGGAGTTCAGACGTGTGCTCTTCCGATCTCACCTTTTCTTCTAAAATCTACAAATCCC
LTC4S	forward	CTCTTTCCCTACACGACGCTCTTCCGATCTTTTTAGGGTTTTGTAGATTTTTATATTATGTTGGAGTTAG
LTC4S	reverse	CTGGAGTTCAGACGTGTGCTCTTCCGATCTCACCCAAAAAACCTTAAACAAATTTCC
TNNI3K	forward	CTCTTTCCCTACACGACGCTCTTCCGATCTATTTTGTGGTTTTTTATAATGTTTTAGGAGTGTGATAA
TNNI3K	reverse	CTGGAGTTCAGACGTGTGCTCTTCCGATCTCCATAATCACTTTATTCACTACATCACCAATACCCATTC
GRM7	forward	CTCTTTCCCTACACGACGCTCTTCCGATCTTTGGGATTATTGTTGATTT
GRM7	reverse	CTGGAGTTCAGACGTGTGCTCTTCCGATCTCCCCTACTACCTACTAAAAATA
CASR	forward	CTCTTTCCCTACACGACGCTCTTCCGATCTTGTAATAGGTATTTGGTTGTAGT
CASR	reverse	CTGGAGTTCAGACGTGTGCTCTTCCGATCTCCCAAACTCTTACTCATTCTA
PRAMEF2	forward	CTCTTTCCCTACACGACGCTCTTCCGATCTTTTGAGGGTATTTAGAAGAGAT
PRAMEF2	reverse	CTGGAGTTCAGACGTGTGCTCTTCCGATCTTCCCTAACTAACTAACTACTAATC
SELP	forward	CTCTTTCCCTACACGACGCTCTTCCGATCTAGAAGGTAGAAAATTAGTAGAGTT
SELP	reverse	CTGGAGTTCAGACGTGTGCTCTTCCGATCTCAACATAAAACTCCATAACTA
CASP14	forward	CTCTTTCCCTACACGACGCTCTTCCGATCTTTGGAGATTTAGTGAGATAATA
CASP14	reverse	CTGGAGTTCAGACGTGTGCTCTTCCGATCTAACAAAACAAATAACCCATATA
KRTAP13.3	forward	CTCTTTCCCTACACGACGCTCTTCCGATCTGAGATTTGTTGGAGGTTTAA
KRTAP13.3	reverse	CTGGAGTTCAGACGTGTGCTCTTCCGATCTCCCAATAAAAAACAACTCC

Supplemental Table S6. Hairpin linkers

Name	Sequence 5' -> 3'	CpG sites [enzyme]
**Hairpin-1**	Phosphate-CTAGCGATGCDDDDDDDDGCATCGCT	*CASR* [AccI]
**Hairpin-2**	Phosphate-TAAGCGATGCDDDDDDDDGCATCGCT	*SELP, PRAMEF2, GRM7, GNAS, DOK6* [CviQI]
**Hairpin-3**	Phosphate-TGAAGCGATGCDDDDDDDDGCATCGCT	*KRTAP13.3* [DdeI]
**Hairpin-4**	Phosphate-AGAGCGATGCDDDDDDDDGCATCGCT	*C12orf12, TNNI3K* [AccI]
**Hairpin-5**	Phosphate-TTAAGCGATGCDDDDDDDDGCATCGCT	*LTC4S* [DdeI]

Hairpin linkers were designed with different overhangs, which are complementary to the sticky ends of the restriction digest at each CpG site with respective enzymes indicated in square brackets. Unique molecular identifiers (UMIs) consisted of random A,G and T nucleotides represented by the letter D.

Supplemental Table S7. Hairpin primers

Primer name	Sequence 5' -> 3'
**HPnewCASR forw**	GTTTAAATTTTTATTTATTTTGTAAGATTTAGG
**HPnewCASR rev**	ACTCTTACTCATTCTACAAAACTC
**HPnewGRM7 forw**	GAGTAGTATGGTTTAGTTGAGG
**HPnewGRM7 rev**	CATAATCCAACTAAAAAAACTACTCC
**HPnewKRTAP13.3 forw**	GTAATTTTTGTTTGATTATGTATGTTGG
**HPnewKRTAP13.3 rev**	ATATTAAATCCAACCCCTACCAC
**HPnewPRAMEF2 forw**	GGTTGGTTGTTGATTAGATGGG
**HPnewPRAMEF2 rev**	CTAACTACTAATCAAATAAACATAACCC
**HPnewSELP forw**	TAGGTAAAGGTTTAGAAAGTGAGG
**HPnewSELP rev**	TAAACAAAAAACAAAAAACCAACAAAATCAC
**HPnewDOK6 forw**	TTAAAGAGATATAATAAAAATAGGTGGG
**HPnewDOK6 rev**	CAAAAAACTACTTAAAATACCTATTTCAC
**HPnewLTC4S forw**	GTTTTTTGTTTTTATTTAGGTTGTTTTTGG
**HPnewLTC4S rev**	ATCCAAACTATTCCTAACAACCC
**HPnewTNNI3K forw**	GTATTATTAGTATTTATTTTTAGTAGAGTG
**HPnewTNNI3K rev**	ATTCACTACATCACCAATACCC
**HPnewGNAS forw**	ATTTTTTTTTTTTGTTTAGAGAGG
**HPnewGNAS rev**	CATCCCTTCTTCTTACTC
**HPnewC12orf12 forw**	AGTTTTAGTTTTATTTAGTATATTTGGG
**HPnewC12orf12 rev**	AATCCCACCCAACACACCT

Supplemental Table S8. 4C primers

Viewpoint	primer	sequence
ALOX12	forward	ATCCAGTAAGGGACAGACAC
ALOX12	reverse	GTAATATCCAAATAAAATGGCTC
LTC4S	forward	GGGTCCTCCCATGGAGAATT
LTC4S	reverse	GAGCACCATGAAGAACTTTGC
KRTAP13.3	forward	CACACACCATTGAAATGACAG
KRTAP13.3	reverse	CTCTGCAATTCTTGCCTGAC
CASR	forward	AGACCGTGACCTTGGCATAG
CASR	reverse	ATGCAGTATTCCACCCTTGC

Supplemental References

1 Koch, C. M. *et al.* Monitoring of cellular senescence by DNA-methylation at specific CpG sites. *Aging cell* **11**, 366-369, doi:10.1111/j.1474-9726.2011.00784.x (2012).

2 Nazor, K. L. *et al.* Recurrent variations in DNA methylation in human pluripotent stem cells and their differentiated derivatives. *Cell stem cell* **10**, 620-634, doi:10.1016/j.stem.2012.02.013 (2012).

3 Weidner, C. I. *et al.* Aging of blood can be tracked by DNA methylation changes at just three CpG sites. *Genome Biology* **15**, R24-R24, doi:10.1186/gb-2014-15-2-r24 (2014).

4 Koch, C. M. *et al.* Pluripotent stem cells escape from senescence-associated DNA methylation changes. *Genome research* **23**, 248-259, doi:10.1101/gr.141945.112 (2013).

5 Olova, N., Simpson, D. J., Marioni, R. E. & Chandra, T. Partial reprogramming induces a steady decline in epigenetic age before loss of somatic identity. *Aging cell* **18**, e12877, doi:10.1111/acel.12877 (2019).

6 Frobel, J. *et al.* Epigenetic Rejuvenation of Mesenchymal Stromal Cells Derived from Induced Pluripotent Stem Cells. *Stem Cell Reports* **3**, 414-422, doi:10.1016/j.stemcr.2014.07.003 (2014).

7 Guelen, L. *et al.* Domain organization of human chromosomes revealed by mapping of nuclear lamina interactions. *Nature* **453**, 948-951, doi:nature06947 [pii];10.1038/nature06947 [doi] (2008).

8 Dixon, J. R. *et al.* Chromatin architecture reorganization during stem cell differentiation. *Nature* **518**, 331-336, doi:10.1038/nature14222 (2015).

9 Kurian, L. *et al.* Conversion of human fibroblasts to angioblast-like progenitor cells. *Nat Methods* **10**, 77-83, doi:10.1038/nmeth.2255 (2013).

10 Reinisch, A. *et al.* Epigenetic and in vivo comparison of diverse MSC sources reveals an endochondral signature for human hematopoietic niche formation. *Blood* **125**, 249-260, doi:10.1182/blood-2014-04-572255 (2015).

11 Fernandez-Rebollo, E. *et al.* Human Platelet Lysate versus Fetal Calf Serum: These Supplements Do Not Select for Different Mesenchymal Stromal Cells. *Scientific Reports* **7**, 5132, doi:10.1038/s41598-017-05207-1 (2017).

12 Schellenberg, A. *et al.* Matrix elasticity, replicative senescence and DNA methylation patterns of mesenchymal stem cells. *Biomaterials* **35**, 6351-6358, doi:S0142-9612(14)00477-3 [pii];10.1016/j.biomaterials.2014.04.079 [doi] (2014).

13 Franzen, J. *et al.* Senescence-associated DNA methylation is stochastically acquired in subpopulations of mesenchymal stem cells. *Aging cell* **16**, 183-191, doi:10.1111/acel.12544 (2017).
