# Supplementary material for: DNA methylation changes during long-term in vitro cell culture are caused by epigenetic drift
Source: Commun Biol. 2021 May 19;4:598. doi: 10.1038/s42003-021-02116-y (PMC8134454; doi:10.1038/s42003-021-02116-y)
Supplement: Supplementary file 3 — Description of Additional Supplementary Files [file 42003_2021_2116_MOESM3_ESM.pdf]

Description of Additional Supplementary Files

File name: Supplementary data 1
Description: Long-term culture-associated CpGs with cutoff $R > 0.7$ or $R < -0.7$.

File name: Supplementary data 2
Description: Pyrosequencing results of validation dataset.

File name: Supplementary data 3
Description: Read numbers of methylation patterns in each amplicon.

File name: Supplementary data 4
Description: Unique molecular identifiers with methylation patterns of hairpin data.
