# Supplementary material for: DNA methylation changes during long-term in vitro cell culture are caused by epigenetic drift
Source: Commun Biol. 2021 May 19;4:598. doi: 10.1038/s42003-021-02116-y (PMC8134454; doi:10.1038/s42003-021-02116-y)
Supplement: Supplementary file 8 — Reporting Summary [file 42003_2021_2116_MOESM8_ESM.pdf]

nature research

Corresponding author(s): Prof. Dr. Dr. Wolfgang Wagner

Last updated by author(s): Mar 25, 2021

Reporting Summary

Nature Research wishes to improve the reproducibility of the work that we publish. This form provides structure for consistency and transparency in reporting. For further information on Nature Research policies, see our Editorial Policies and the Editorial Policy Checklist.

Statistics

For all statistical analyses, confirm that the following items are present in the figure legend, table legend, main text, or Methods section.

n/a	Confirmed	
☐	☒	The exact sample size (n) for each experimental group/condition, given as a discrete number and unit of measurement
☐	☒	A statement on whether measurements were taken from distinct samples or whether the same sample was measured repeatedly
☐	☒	The statistical test(s) used AND whether they are one- or two-sided<br>*Only common tests should be described solely by name; describe more complex techniques in the Methods section.*
☒	☐	A description of all covariates tested
☐	☒	A description of any assumptions or corrections, such as tests of normality and adjustment for multiple comparisons
☐	☒	A full description of the statistical parameters including central tendency (e.g. means) or other basic estimates (e.g. regression coefficient) AND variation (e.g. standard deviation) or associated estimates of uncertainty (e.g. confidence intervals)
☐	☒	For null hypothesis testing, the test statistic (e.g. F, t, r) with confidence intervals, effect sizes, degrees of freedom and P value noted<br>*Give P values as exact values whenever suitable.*
☒	☐	For Bayesian analysis, information on the choice of priors and Markov chain Monte Carlo settings
☒	☐	For hierarchical and complex designs, identification of the appropriate level for tests and full reporting of outcomes
☐	☒	Estimates of effect sizes (e.g. Cohen's d, Pearson's r), indicating how they were calculated

Our web collection on statistics for biologists contains articles on many of the points above.

Software and code

Policy information about availability of computer code

Data collection	*Provide a description of all commercial, open source and custom code used to collect the data in this study, specifying the version used OR state that no software was used.*
Data analysis	R packages: stats, impute, lumi, car, leaps, gplots, caret, ggplot2, RCircos<br>python packages: scipy, seaborn<br>FastQC<br>TrimGalore<br>Bismark<br>bowtie2<br>4Cker<br>DESeq2<br>MACS2<br>Deeptools<br>RGT motif analysis

For manuscripts utilizing custom algorithms or software that are central to the research but not yet described in published literature, software must be made available to editors and reviewers. We strongly encourage code deposition in a community repository (e.g. GitHub). See the Nature Research guidelines for submitting code & software for further information.

Data

Policy information about availability of data

All manuscripts must include a data availability statement. This statement should provide the following information, where applicable:

- Accession codes, unique identifiers, or web links for publicly available datasets
- A list of figures that have associated raw data
- A description of any restrictions on data availability

Raw data of DNA methylation and hydroxymethylation profiles generated in this study and raw data as well as processed data of CTCF ChIP-seq and 4C-seq analysis were submitted to Gene Expression Omnibus (GEO): GSE144196

Field-specific reporting

Please select the one below that is the best fit for your research. If you are not sure, read the appropriate sections before making your selection.

- [x] Life sciences
- [ ] Behavioural & social sciences
- [ ] Ecological, evolutionary & environmental sciences

For a reference copy of the document with all sections, see nature.com/documents/nr-reporting-summary-flat.pdf

Life sciences study design

All studies must disclose on these points even when the disclosure is negative.

Sample size	No sample size calculation was performed. Sample sizes were determined based on feasibility for each experiment.
Data exclusions	Culture-associated CpGs: An outlier test using the R package car was performed resulting in exclusion of one sample (GSM1004625).<br>Analysis of hemimethylation: One late passage donor of CASR is missing due to failed sequencing.
Replication	All experiments were performed for at least two independent cell donors and at at least four culture-associated CpGs.
Randomization	Randomization was not performed.
Blinding	Blinding was not performed.

Reporting for specific materials, systems and methods

We require information from authors about some types of materials, experimental systems and methods used in many studies. Here, indicate whether each material, system or method listed is relevant to your study. If you are not sure if a list item applies to your research, read the appropriate section before selecting a response.

Materials & experimental systems

n/a	Involved in the study	
[ ]	[x]	Antibodies
[x]	[ ]	Eukaryotic cell lines
[x]	[ ]	Palaeontology and archaeology
[x]	[ ]	Animals and other organisms
[ ]	[x]	Human research participants
[x]	[ ]	Clinical data
[x]	[ ]	Dual use research of concern

Methods

n/a	Involved in the study	
[ ]	[x]	ChIP-seq
[x]	[ ]	Flow cytometry
[x]	[ ]	MRI-based neuroimaging

Antibodies

Antibodies used	ChIP-validated CTCF antibody (Active Motif)
Validation	ChIP-validation was provided by the manufacturer

Human research participants

Policy information about studies involving human research participants

Population characteristics	*Describe the covariate-relevant population characteristics of the human research participants (e.g. age, gender, genotypic information, past and current diagnosis and treatment categories). If you filled out the behavioural & social sciences study design questions and have nothing to add here, write "See above."*
Recruitment	*Describe how participants were recruited. Outline any potential self-selection bias or other biases that may be present and how these are likely to impact results.*
Ethics oversight	All human cell samples were taken after informed and written consent was obtained from donors and the study was specifically approved by the ethics committees of RWTH Aachen University Medical School (permit numbers: EK300/13, EK163/07, EK 187/08), University of Heidelberg, and Hannover Medical School.

Note that full information on the approval of the study protocol must also be provided in the manuscript.

ChIP-seq

Data deposition

- [x] Confirm that both raw and final processed data have been deposited in a public database such as GEO.
- [x] Confirm that you have deposited or provided access to graph files (e.g. BED files) for the called peaks.

Data access links
May remain private before publication.

https://www.ncbi.nlm.nih.gov/geo/query/acc.cgi?acc=GSE144196

Files in database submission

BM168_P2_norm.bw
BM170_P2_norm.bw
OB4C_P2_norm.bw
BM168_P8_norm.bw
BM170_P14_norm.bw
OB4C_P14_norm.bw
Input_early_norm.bw
Input_late_norm.bw
combined_peaks_with_CTCF_motif.bed
combined_peaks_with_CTCF_motif_early.bed
combined_peaks_with_CTCF_motif_late.bed
BM168_P2_S5_R1_001.fastq.gz
BM170_P2_S3_R1_001.fastq.gz
OB4C_P2_S7_R1_001.fastq.gz
BM168_P8_S6_R1_001.fastq.gz
BM170_P14_S4_R1_001.fastq.gz
OB4C_P14_S8_R1_001.fastq.gz
Input_early_S1_R1_001.fastq.gz
Input_late_S2_R1_001.fastq.gz
BM168_P2_S5_R2_001.fastq.gz
BM170_P2_S3_R2_001.fastq.gz
OB4C_P2_S7_R2_001.fastq.gz
BM168_P8_S6_R2_001.fastq.gz
BM170_P14_S4_R2_001.fastq.gz
OB4C_P14_S8_R2_001.fastq.gz
Input_early_S1_R2_001.fastq.gz
Input_late_S2_R2_001.fastq.gz

Genome browser session
(e.g. UCSC)

Provide a link to an anonymized genome browser session for "Initial submission" and "Revised version" documents only, to enable peer review. Write "no longer applicable" for "Final submission" documents.

Methodology

Replicates	Three biological replicates were used both in early and late passages.
Sequencing depth	Reads approximately exhibited a 97% concordant alignment rate in all samples and sequencing depth ranged from 38 to 170 million reads.
Antibodies	ChIP-validated CTCF antibody (Active Motif)
Peak calling parameters	We used MACS2 for peak calling on each sample with the default parameters against the input control.

Data quality	*Describe the methods used to ensure data quality in full detail, including how many peaks are at FDR 5% and above 5-fold enrichment.*
Software	MACS2, Deeptools, bowtie2, RGT motif analysis, R stats
